# Simulation of overland flow considering the influence of topographic depressions

**DOI:** 10.1038/s41598-020-63001-y

**Published:** 2020-04-09

**Authors:** Lin Hu, Weimin Bao, Peng Shi, Jianjin Wang, Meixia Lu

**Affiliations:** 1 Department of Hydrology and Water Resources, University of Hohai, Nanjing, 210098 China; 2 State Key Laboratory of Hydrology-Water Resources and Hydraulic Engineering, Hohai University, Nanjing, 210098 China; 3 Zhejiang Institute of Hydraulics and Estuary, Hangzhou, 310020 China

**Keywords:** Hydrology, Hydrogeology

## Abstract

The simulation of overland flow, wherein runoff yield and concentration are influenced by topography, is fundamental to hydrological forecasting. Therefore, critically evaluating the characteristics of overland flow under the influence of topographic depressions—which are one of the most common microtopographic structures—is vital for improving current hydrological models. In this study, we developed a solution for the real-world application of overland flow simulations under the influence of depressions in hydrological models. A relative depression storage–outflow curve (RDOC) was proposed to investigate surface outflow processes. Experiments were conducted based on the variable-controlling approach using three rainfall return periods, four slopes, and four depression rates while ensuring a consistent initial soil moisture content. A homogenized RDOC was achieved based on shape analysis; it was parameterized by the outflow threshold and the reciprocal of the curve index of two outflow stages (B and D). A relative depression storage–outflow function (RDOF) was generated and a complete calculation procedure was applied within a hydrological model. Furthermore, we analyzed the hydrological responses to parameters of different hydrological factors to improve our understanding of the parameter determination of the RDOF.

## Introduction

Flood disasters are among the most destructive natural disasters faced by humans. They greatly threaten the safety, lives, and property of people worldwide^1–3^. To further improve the accuracy of flood forecasting, the effects of microtopography on hydrological responses have become a key global research topic^4–6^. As one of the most common microtopographic structures, depressions are widely distributed across all types of soils, vegetation, and rocks, and even occur as mountain ditches^7^; these structures significantly affect the runoff yield and concentration^8^. During a flood event, when rainfall exceeds evaporation and infiltration, runoff in a basin with no depressions will move directly along the slope towards a river or outlets. However, when depressions exist, water exceeding the evaporation and infiltration capacity will first be stored within these topographic lows. The water stored in depressions will not spill over until it reaches the depression storage threshold, after which the rainfall in depressions and the incoming water from upstream will enter the spillover stage. The confining influence of the rough surface gradually decreases with the increasing formation of overland flow, which greatly facilitates the process of runoff confluence. Thus, the spatial structure of topographic depressions has a significant redistributing effect on the rainfall–runoff process over time. It is of great practical value to further explore the influence of depression impoundment on overland flow characteristics, as doing so allows for the construction of more accurate flood models. Furthermore, the results of this study will enhance the accuracy of simulated overland flow in regions with numerous depressions.

Previous studies on the microtopography of depressions have predominately focused on the effects of their fundamental characteristics on hydrological processes. For example, depressions can delay the initial time of runoff yield^9^, as well as the volume of outflow^10^. The existence of depressions increases the roughness of soil surfaces^11^ and reduces the velocity of overland flow^12^. Surface depressions enhance the retention of runoff water^13^ and the depression storage areas behave as temporary passive storages of overland flow, resulting in delays in the hydrograph signals^14^. Maximum depression storage has previously been estimated from different hydrological factors^15,16^, and the interaction between topographic depressions and infiltration has also been explored^17^. In recent years, studies on depressions have focused on the functional and structural features^18^ related to the appearance of overland flow. The concept of hydrological connectivity has been used to express these features and has been studied in a variety of disciplines^19–21^. To obtain effective indicators of the connectivity properties, Antoine *et al*. (2009) conducted numerical experiments and proposed the relative surface connection function (RSCF) as a functional connectivity indicator^22^. Additionally, Peñuela *et al*. (2015) characterized the RSCF and estimated the extent to which it can be predicted by structural indicators^23^. To characterize the runoff generation process and the related spatiotemporal variations, Yang and Chu (2013) proposed a conceptual puddle-to-puddle model to track the evolution of connectivity and simulate outflow^24^. Meanwhile, methods based on high-resolution digital elevation models (DEMs) are not yet applicable in areas for which there is insufficient data^25^.

In contrast to previous studies, this work was conducted to develop a solution for the application of overland simulations under the influence of topographic depressions in hydrological models. This study is mainly focused on the relationship between surface outflow and the water stored in depressions, rather than any specific overland flow generative mechanism. Therefore, our primary goal was to construct a convenient function to express the outflow process. To achieve this, we set four specific objectives: (1) to introduce the concept of a relative depression–outflow curve (RDOC) and establish its relationship to the simulation of overland flow; (2) to analyze the shape of RDOCs modelled with different hydrological factors; (3) to construct a function based on the curve and complete its calculation as applied in a hydrological model; (4) to analyze the hydrological responses to function parameters of different slopes and rainfall intensities.

## Materials and Methods

### Relative depression storage–outflow curve (RDOC)

**Figure 1:**
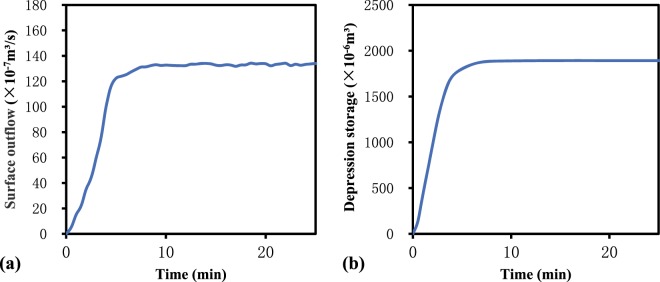
Time-variant curve of (**a**) overland flow and (**b**) depression storage.

**Figure 2:**
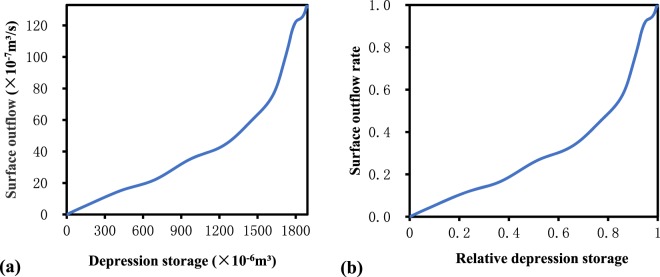
(**a**) Depression storage–outflow curve; (**b**) relative depression storage–outflow curve (RDOC).

During a rainfall event, the overland flow (Fig. 1a) and depression storage processes (Fig. 1b) generally begin at the same time, and both first increase and then stabilize. By excluding the influence of time, we simultaneously combined the surface outflow with the depression storage. The growth of depression storage was then selected for visualization (Fig. 2a). Regarding the building ideal of the RSCF proposed by Antoine *et al*.^20^, we normalized the abscissa and ordinate to eliminate the influence of the time dimension on the shape of the curve (Fig. 2b).

The curves built in this study are RDOCs. The difference between the RSCF and RDOC is their ordinates. The ordinate of the RSCF is the area connected to the outlet, which is equal to the R/P (Runoff/Precipitation) when surface confinement is ignored. The ordinate of the RDOC is the relative surface outflow rate, which is expressed as R_out_/(P-E-*i*), and is equivalent in numerical value to R_out_/R_yield_ when ignoring surface confinement (i.e., flow roughness), where R_out_ is the surface outflow (mm), R_yield_ is the runoff yield (mm), E is the evaporation (mm), and *i* is the infiltration (mm). This data represents the cumulative number of outflow readings obtained every 30 s. Here, R_yield_ corresponds to the value of rainfall minus evaporation and infiltration. In this experiment, the amount of evaporation relative to water storage is negligible. For infiltration, this experiment guarantees that the initial soil moisture content of each experiment is like that of the soil tester and is consistent with the initial soil moisture content of the non-depression experiment. This implies that at each moment of the experiment the same rainfall and rainfall intensity is experienced. When the slope is different, the infiltration amount of each group of control experiments corresponding to different depression rates is the same. The infiltration amount is subtracted from the outflow result of the non-depression experiment to obtain R_yield_. Determining the function of the RDOC is of great importance to minimize the effect of the lack of a depression calculation module on the runoff yield and concentration modules when simulating overland flow in areas with depressions.

### Experimental settings

**Figure 3:**
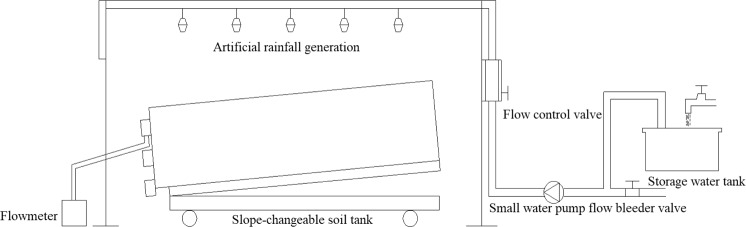
Schematic of the experimental instruments.

**Figure 4:**
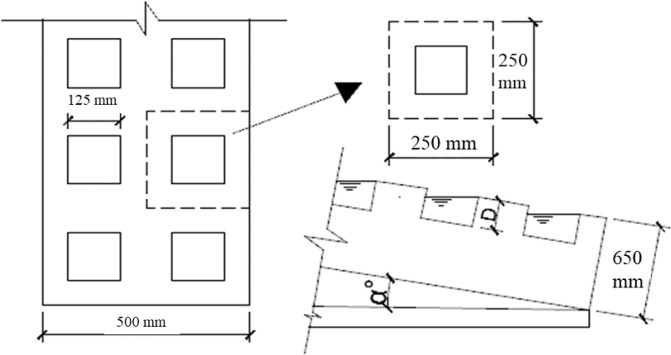
Local schematic map of standardized depressions.

**Figure 5:**
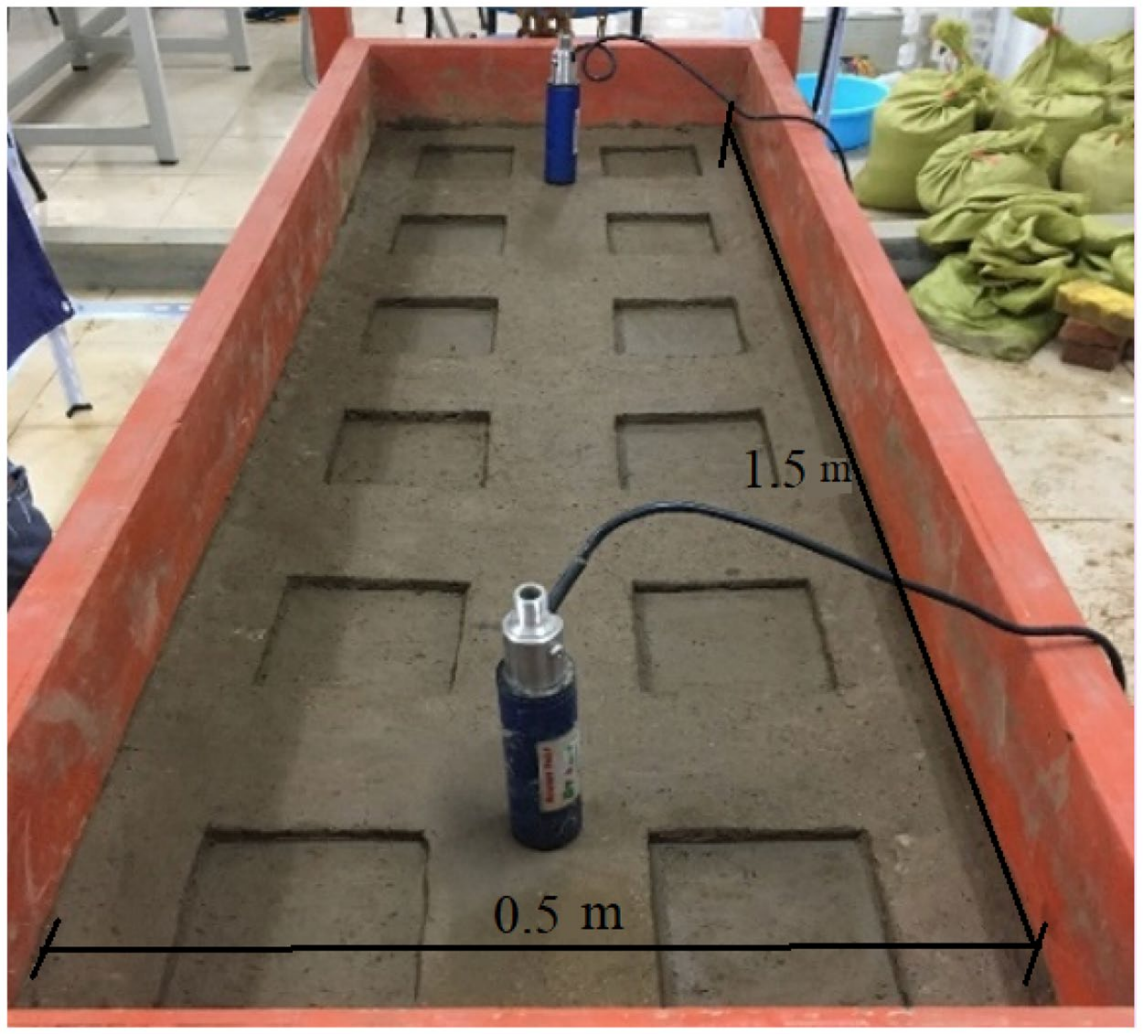
Physical display of constructed depressions in the slope-adjustable soil tank.

Experiments were conducted in the runoff yield and concentration laboratory of Hohai University, Nanjing, China. The experimental instruments, including an artificial rainfall generator and slope-changeable soil tank, are shown in Fig. 3. The dimensions of the soil tank were 1.5 m × 0.5 m × 0.65 m. For simulating outflow, standardized depressions were constructed on the soil trough. The surface area of each depression was a square with dimensions of 0.125 m × 0.125 m. To ensure that the depressions met the experimental requirements of size, location, and depth, they were built using a template made of wood. The depression layout is shown in Fig. 4, and the completed depressions are shown in Fig. 5.

In this study, typical tillage soil from Nanjing was used as the research material. The original soil material recovered was analyzed to determine its physicochemical properties, and all analyses were averaged after four repetitions. The soil density and field water holding capacity were determined by ring shear testing recommended by the agricultural industry-standard (NY/T 1121.22-2010), issued by the Ministry of Agriculture of the People’s Republic of China. The results showed that the dry density of the soil was 1.58 g/cm and the field water holding capacity was 214.59 g/kg. Grain size analysis was performed using an LS13320 Laser Diffraction Particle Size Analyzer (Beckman Coulter Inc., USA). The measured clay content (<0.002 mm) accounted for 12.11% (0.02–0.002 mm), the silt content accounted for 50.2% (2–0.02 mm), and the sand content accounted for 37.69% of the soil. According to the international classification standard for soil adopted at the 2^nd^ International Soil Society meeting, the soil texture is silty loam.

The experimental soil pretreatment process primarily included an air-drying treatment and grinding screening, and the treated soil utilization rate exceeded 80%. Before filling, a 5-cm layer of soil was placed in each tank, and a layered filling was used. The fill volume of each 5-cm layer was calculated according to the measured soil density, and the weighed soil was poured into the soil trough, which was filled with steamed bread after the soil was filled. To prevent soil stratification, the soil was gently shaved with a shovel, and the soil layer was tightly combined. After the filling was completed, the surface runoff of the earthen trough and the outflow nozzle of the soil were blocked, and only the underground diameter outflow nozzle was reserved to fill the surface of the soil; it was subsequently slowly infiltrated. After a few days, the soil surface water was reduced, and water was continuously poured until the water outlet of the underground channel of the tank flowed out. The soil inside the tank was saturated at this time.

After the soil moisture in the trough had completely infiltrated, the soil was allowed to stand to reduce the water content and restore its natural state. Every two to three days, the soil surface was lightly watered to prevent the soil surface from cracking, and to stop the soil water content from falling too low. This process lasted for two months. After the soil in the tank had settled for more than two months, subsequent experiments were performed.

To fully explore the hydrological response of RDOCs under different hydrological factors, we conducted this study with three return periods, four slopes, and four depression depths (Table 1).

**Table 1:** Experimental variable settings.

Return period (year)	2	5	10	
Slope (°)	2.5°	5°	7.5°	10°
Depression depth (cm)	0.5	1	1.5	2

The rainfall intensity at 2-year, 5-year, and 10-year return periods in Nanjing was 0.824 mm/min, 1.044 mm/min, and 1.209 mm/min, respectively. Considering that the depression depth represents the only attribute of each depression and the remaining variables were representative of the overall nature, the depression depth was replaced with the depression rate (DR), which can be considered as the increase in the surface area when the depression is increased, compared to when there is no depression. The DR is calculated as follows:

1 $${\rm{DR}}=({{\rm{S}}}_{{\rm{surface}}}/{{\rm{S}}}_{{\rm{projection}}}-1)\times 100 \% $$

where S_surface_ is the surface area of the entire slope, including the depressions, and S_projection_ is the projection area perpendicular to the direction of the slope. The resultant depression rates of the standardized depressions constructed in this experiment are listed in Table 2.

**Table 2:** Depression rates of standardized depressions.

Depression depth (cm)	0	0.5	1	1.5	2
Depression rate (%)	0	4	8	12	16

### Experimental procedure

**The main experimental procedure was as follows:**
1. Standardized depressions of specified depths were constructed on the surface of the soil and the slope was adjusted to a specified gradient.
2. A pre-rainfall period was conducted before the start of the experiment to eliminate the effects of the initial soil water contents. Soil monitoring instruments were used to ensure that the soil had been saturated, artificial rainfall was stopped, and the surface water of the soil was cleared.
3. The artificial rainfall was adjusted to generate rainfall of a specified intensity, and a waterproof cloth was used to prevent infiltration of the heterogeneous initial rainfall that occurs in the first 5 min into the soil.
4. Surface outflow was recorded every 30 s after the waterproof cloth was removed.
5. Each experiment was repeated three times, and the averages were taken as the experimental results.

## Results and Discussion

In this study, the shape of the RDOCs was first analyzed under different hydrological conditions, followed by construction of the function based on these analyses. To facilitate the application of the function to actual hydrological simulations, the establishment of function parameter values and the hydrological responses of different factors were further studied.

### Curve shape analysis of the relative depression storage–outflow process

**Figure 6:**
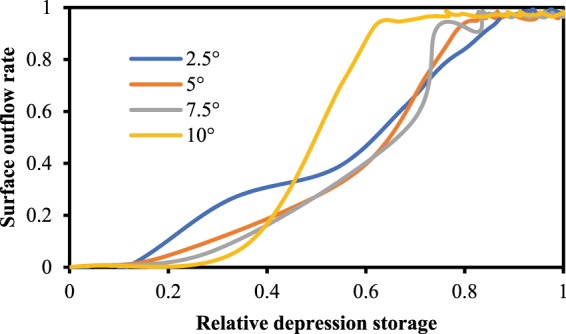
RDOCs of different slopes when DR = 4% and the rainfall return period is 5 years. Each curve represents the average of three replicates.

To construct an RDOC, the surface outflow and depression storage of each moment are required. The surface outflow was determined from the discharge collected every 30 s, and the depression storage was calculated from the difference between the surface outflow curves under the depression and without depressions. Observing the RDOCs constructed by this method, we found that when DR ≤ 4%, the end-point judgment of depression storage was problematic in some cases (e.g., Fig. 6). The reason for this error is mainly due to the surface outflow exhibiting a certain degree of volatility. The influence of DR ≤ 4% on the surface outflow is relatively small compared to the surface outflow volatility when the depressions are close to the spillover point. Therefore, only the results for DR > 4% were used in subsequent analyses.

**Figure 7:**
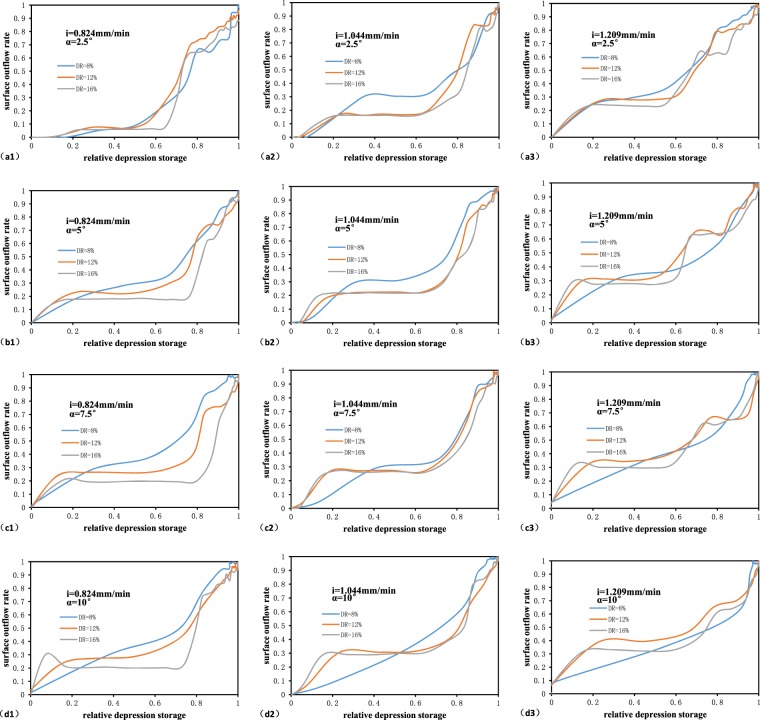
RDOCs constructed under different hydrological factors. Each curve is the average of three replicates. Each column (**a–d**) has a single slope (*α*º) and each row (a1–a3; b1–b3; c1–c3; d1–d3) has a single rainfall intensity (*i* mm/min).

The RDOCs constructed under different hydrological factors are summarized according to their different depression rates in Fig. 7. The results indicate that although there were differences in the RDOCs under different hydrological factors, there was no obvious difference in their shapes under the same rainfall intensity and slope. The RDOCs with distinct differences in shape were c2, d2, and d3, in which DR = 8%. This is mainly due to the relatively small depression rate, sleep stope, and high rainfall intensity. The RDOCs have a second flat stage in their curves (Fig. 7. a3, b3, and c3) when the rainfall intensity is higher, particularly when this is accompanied by an elevated depression rate is. The flat stage also exists when the rain intensity is low. This is because the initial relative surface depression rate decreases in the flat stage after the rain intensity increases, magnifying the observation on the figure. This shows that when the rain intensity is large enough, the increased rate of the relative depression storage rate is higher than that of the relative surface outflow rate, i.e., the increase rate of the water storage rate is higher. Each of these factors can weaken the effect of depressions to some extent, and the combination of all three factors determined the original shape of the RDOC.

**Figure 8:**
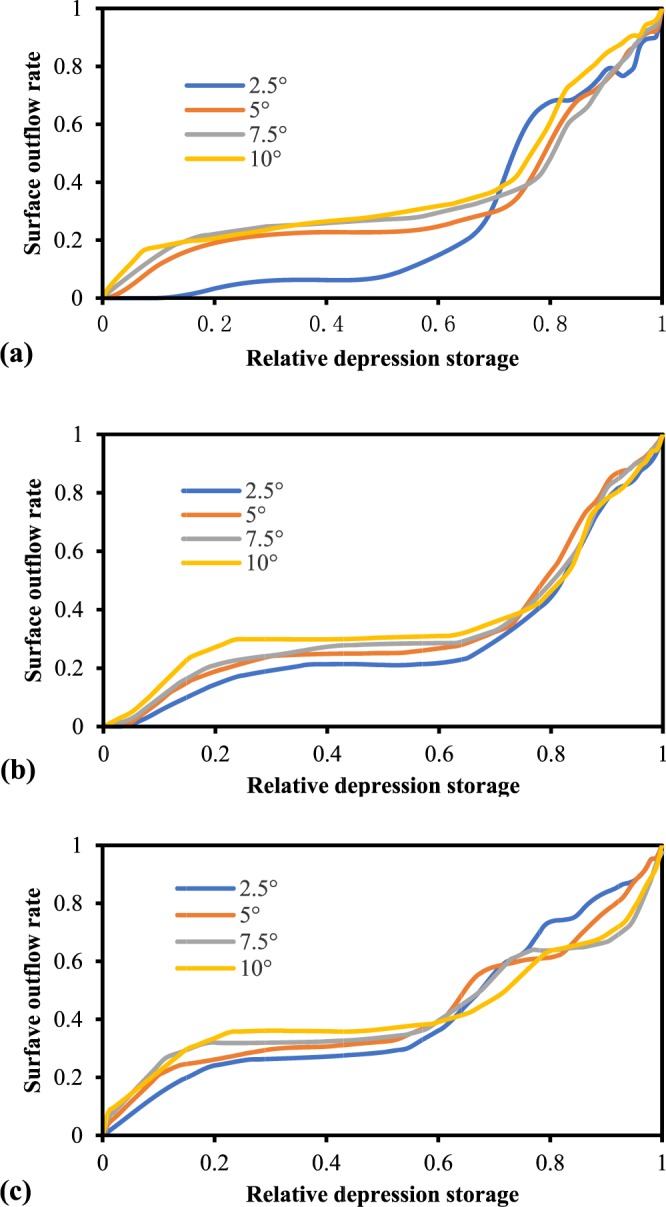
Homogenized RDOCs of different slopes with a rainfall return period of (**a**) 2 years (0.824 mm/min), (**b**) 5 years (1.044 mm/min), and (**c**) 10 years (1.209 mm/min). Each curve is homogenized by three depression rates and is the average of three replicates.

By homogenizing the depression rate, updated RDOCs were generated; these are shown in Fig. 8 after excluding the curves c2, d2, and d3. The results suggest that the shape of RDOCs under different rainfall intensities are generally similar. The only exception to this pattern was the RDOC with a return period of 2 years and a slope of 2.5°, whose surface outflow rate did not markedly increase until the relative depression storage reached ~0.1. This is mainly because the rainfall intensity with a return period of 2 years was low. During the initial stage, a considerable portion of the rainfall was used for wetting the soil when the rainfall intensity was low, and therefore the surface outflow was slow and not easily obtained by the method used in our calculations. Our experiments also revealed that as the slope increases, the effective storage capacity of the depression declines. Since the water in the depression is affected by gravity, an increase in slope causes some of the water to overflow from the depression. Under extreme conditions, when the slope reaches 90 degrees, the depression cannot store water.

**Figure 9:**
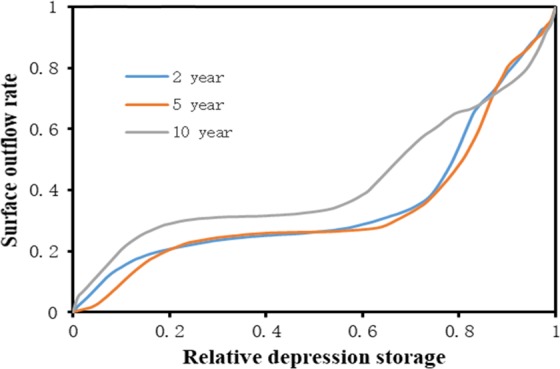
Homogenized RDOCs of different rainfall return periods. Each curve is homogenized by three depression rates and four slopes and represents the average of three replicates.

**Figure 10:**
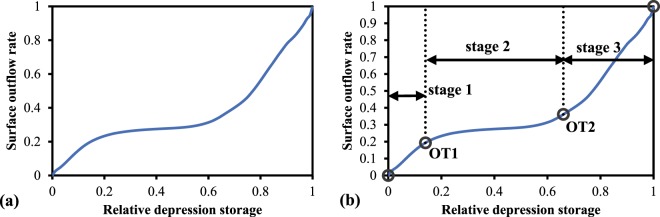
Homogenized RDOC: (**a**) original and **(b**) after stage partitioning.

After the elimination of one outlier curve, the remaining RDOCs of the same slope were homogenized (Fig. 9). After homogenization, the shape of the RDOCs still exhibited slight differences between the rainfall events of the different return periods. These results imply that homogenization is an effective method of processing RDOCs. Furthermore, the RDOCs of the same rainfall intensity were also homogenized, and a final average RDOC was obtained (Fig. 10a). As shown in Fig. 10a, the shape of the RDOC first increases rapidly and then slows gradually. After slowly rising again, it then reaches a steeper gradient and the surface outflow rate approaches one.

To more accurately divide the stages of the RDOC, the outflow threshold was adopted in reference to the connected threshold used by Peñuela *et al*.^23^. The outflow threshold occurred when the rate of increase of the surface outflow was equal to the excess relative depression storage (i.e., the slope of the curve was equal to one). Using this method, two outflow threshold points, OT1 and OT2 (Fig. 10b) were obtained, and the overall RDOC process was divided into three stages.

In this study, the three partitioned stages were defined as the (1) surface-wetting stage, (2) gentle rising stage, and (3) rapid rising stage. During the surface wetting stage, the growth rate of surface outflow exceeded that of depression storage. This was possibly the result of the convergence of accumulated water on the surface resulting in a rapid flow moving toward the boundary of the soil trough. In the gentle rising stage, the relative depression storage increased significantly more than the surface outflow rate. From a theoretical viewpoint, the volume of water stored in depressions should gradually increase, while the surface outflow rate typically remains unchanged in this stage. However, under actual conditions, the surface outflow rate also increased slightly. This may be due to the soil crust caused by water scouring, which led to a decrease in the infiltration rate and an increase in the surface outflow. Furthermore, it accelerated the speed of the surface outflow, as surface roughness was reduced. Finally, during the rapid rise stage, the surface outflow rate increased rapidly because the volume of water stored in the depressions reached the maximum storage capacity and spilled out. Theoretically, all depressions should reach the spillover point simultaneously. However, uneven distribution of rainfall and the uncertainty of the flow paths of overland flow caused the respective spillover points to differ by variable degrees. Additionally, spillover occurred before the maximum depression storage was reached, and contributed to the corresponding relative depression storage of OT2 being less than one.

### Construction of the relative depression storage–outflow function (RDOF)

**Figure 11:**
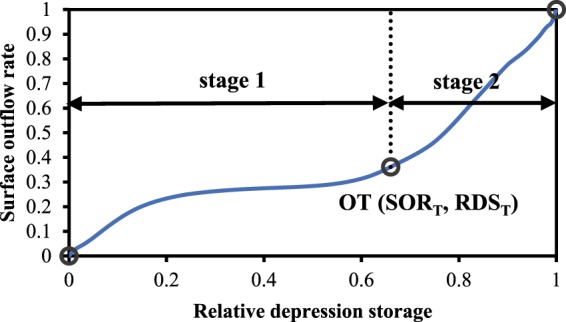
Schematic of the stage division in RDOF construction.

To apply the RDOC to the calculations of a hydrological model, it is necessary to express the curve as an RDOF. Considering the complexity of the shape of the RDOC, we combined the first two stages and solved the curve-fitting step-by-step. In this study, we defined the two stages after integration as the slow rising stage and the fast-rising stage (Fig. 11). The junctional point of these stages was then defined as the outflow threshold, which is equal to the original OT2.

The formulae for the surface outflow rate (SOR) and relative depression storage (RDS) are as follows:

2 $${\rm{SOR}}=\frac{{{\rm{R}}}_{{\rm{out}}}}{{{\rm{R}}}_{yield}};$$

3 $${\rm{RDS}}=\frac{{\rm{DS}}}{{\rm{DSM}}},$$

where R_out_ is the surface outflow (mm), R_yield_ is the runoff yield (mm), DS is the depression storage (mm), and DSM is the maximum depression storage (mm). These equations were formulated in reference to the parabolic equation used to construct the storage capacity curve^26^. The equation for the first stage is expressed as follows:

4 $$\frac{{\rm{SOR}}}{{{\rm{SOR}}}_{{\rm{T}}}}=1-{\left(1-\frac{{\rm{RDS}}}{{{\rm{RDS}}}_{{\rm{T}}}}\right)}^{\frac{1}{{\rm{B}}}}\,{,{\rm{RDS}} < {\rm{RDS}}}_{{\rm{T}}},$$

where SOR_T_ is the surface outflow rate corresponding to the outflow threshold point, RDS_T_ is the relative depression storage corresponding to the outflow threshold point, and B is the reciprocal of the curve index of the slow rising stage. Similarly, the equations for the second stage is expressed as follows:

5 $$\frac{{{\rm{SOR}}-{\rm{SOR}}}_{{\rm{T}}}}{1{-{\rm{SOR}}}_{{\rm{T}}}}=1\,-\,{\left(1-\frac{{{\rm{RDS}}-{\rm{RDS}}}_{{\rm{T}}}}{1{-{\rm{RDS}}}_{{\rm{T}}}}\right)}^{\frac{1}{{\rm{D}}}},RDS\ge {{\rm{RDS}}}_{{\rm{T}}},$$

where D is the reciprocal of the curve index of the fast-rising stage. Combining Eqs. 4 and 5, the formula for the SOR can be expressed as follows:

6 $${\rm{SOR}}=\{\begin{array}{l}{{\rm{SOR}}}_{{\rm{T}}}\times \left(1\,-\,{\left(1-\frac{{\rm{RDS}}}{{{\rm{RDS}}}_{{\rm{T}}}}\right)}^{\frac{1}{{\rm{B}}}}\right),\,{{\rm{RDS}} < {\rm{RDS}}}_{{\rm{T}}}\\ (1{-{\rm{SOR}}}_{{\rm{T}}})\times \left(1\,-\,{\left(1-\frac{{{\rm{RDS}}-{\rm{RDS}}}_{{\rm{T}}}}{1{-{\rm{RDS}}}_{{\rm{T}}}}\right)}^{\frac{1}{{\rm{D}}}}\right){+{\rm{SOR}}}_{{\rm{T}}},\,{\rm{RDS}}\ge {{\rm{RDS}}}_{{\rm{T}}}\end{array}.$$

When applied in a hydrological model, Eq. 6 can be inserted between the surface runoff yield and the concentration processes that account for the effects of depressions. The actual surface outflow under the influence of depressions at a given moment may then be calculated. The DSM parameter can be obtained through digital elevation model (DEM) processing or estimated using local empirical formulae. The amount of water stored in depressions in the initial state (DS_0_) can be estimated by local conditions. For example, in the long-term and rain-free season, the DS_0_ may be close to zero. Therefore, the relative depression storage in the initial state (RDS_0_) can be calculated by Eq. 7, as follows:

7 $${{\rm{RDS}}}_{0}=\frac{{{\rm{DS}}}_{0}}{{\rm{DSM}}}.$$

By substituting the calculated RDS_0_ into Eq. 6, the formula for calculating the SOR at the initial moment is obtained:

8 $${{\rm{SOR}}}_{0}=\{\begin{array}{l}{{\rm{SOR}}}_{{\rm{T}}}\times \left(1\,-\,{\left(1-\frac{{{\rm{RDS}}}_{0}}{{{\rm{RDS}}}_{{\rm{T}}}}\right)}^{\frac{1}{{\rm{B}}}}\right),\,{{\rm{RDS}}}_{0}{ < {\rm{RDS}}}_{{\rm{T}}}\\ (1{-{\rm{SOR}}}_{{\rm{T}}})\times \left(1\,-\,{\left(1-\frac{{{\rm{RDS}}}_{0}{-{\rm{RDS}}}_{{\rm{T}}}}{1{-{\rm{RDS}}}_{{\rm{T}}}}\right)}^{\frac{1}{{\rm{D}}}}\right){+{\rm{SOR}}}_{{\rm{T}}},\,{{\rm{RDS}}}_{0}\ge {{\rm{RDS}}}_{{\rm{T}}}\end{array}.$$

Furthermore, when the SOR_0_ and R_yield_ of the initial state are combined, the R_out_ at the current moment can be calculated as follows:

9 $${{\rm{R}}}_{{\rm{out}}}{={\rm{R}}}_{{\rm{yield}}}\times {{\rm{SOR}}}_{0}.$$

The depression storage of the next moment (DS′) may then be updated using Eq. 9 to produce:

10 $${\rm{D}}{\rm{S}}{\prime} =\frac{{{\rm{DS}}}_{0}{+{\rm{R}}}_{{\rm{yield}}}{-{\rm{R}}}_{{\rm{out}}}}{{\rm{DSM}}}.$$

Finally, the updated depression storage may be used to update the relative depression storage (RDS′) and surface outflow rate (SOR′) of the next moment as follows:

11 $${\rm{RD}}{\rm{S}}{\prime} =\frac{{\rm{D}}{\rm{S}}{\prime} }{{\rm{DSM}}};$$

12 $${\rm{SO}}{\rm{R}}{\prime} =\{\begin{array}{l}{{\rm{SOR}}}_{{\rm{T}}}\times \left(1\,-\,{\left(1-\frac{{\rm{RD}}{\rm{S}}{\prime} }{{{\rm{RDS}}}_{{\rm{T}}}}\right)}^{\frac{1}{{\rm{B}}}}\right),\,{{\rm{RD}}{\rm{S}}{\prime}  < {\rm{RDS}}}_{{\rm{T}}}\\ (1{-{\rm{SOR}}}_{{\rm{T}}})\times \left(1\,-\,{\left(1-\frac{{{\rm{RD}}{\rm{S}}{\prime} -{\rm{RDS}}}_{{\rm{T}}}}{1{-{\rm{RDS}}}_{{\rm{T}}}}\right)}^{\frac{1}{{\rm{D}}}}\right){+{\rm{SOR}}}_{{\rm{T}}},{\rm{RD}}{\rm{S}}{\prime} \ge {{\rm{RDS}}}_{{\rm{T}}}\end{array}.$$

These processes are then repeated to calculate the results for all the periods.

When applying Eq. 9 to calculate the R_out_, the R_yield_ is generally used as the runoff yield at the initial moment of the period. The surface outflow and runoff yield processes occur simultaneously and continuously. This leads to the introduction of a forward difference error. To reduce the effect of this error, it is necessary to take 5 mm as the basic unit of runoff yield, and R_yield_ must be divided into multiple units. By repeatedly using Eqs. 9–12 to calculate the R_out_ of each unit, the final R_out_ of a period can be obtained.

### Analysis of hydrological responses to RDOF parameters

**Figure 12:**
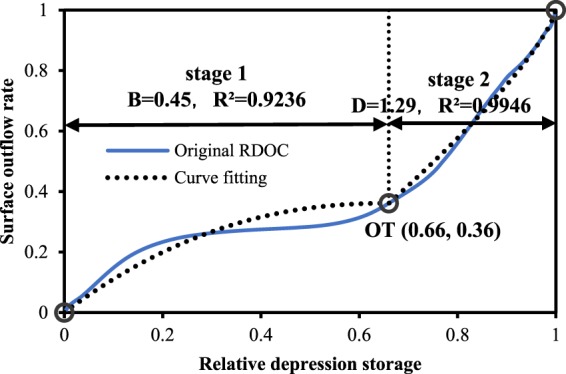
Characteristic parameters of the homogenized RDOF.

To better apply the RDOF to real-world hydrological forecasting, it is necessary to critically evaluate the parameters of the RDOF. To this end, the homogenized parameters of the RDOF were first calculated; these can be directly used in an area with no data or as initial parameters before further calibration. Secondly, the hydrological responses to the RDOF parameters were analyzed. These responses can provide insight into how best to adjust the parameters based on the local slope, depression rate, and dominant rainfall intensity. For this purpose, the initial values of the homogenized RDOF parameters (Fig. 11) were computed and are visualized in Fig. 12. As shown in Fig. 12, the relative depression storage corresponding to the outflow threshold was 0.66, and the surface outflow rate was 0.36. To characterize the degree of fit, we used the Levenberg-Marquardt method and general global optimization methods for calculation. When the parameter B is 0.45, the square of the correlation coefficient in the slowly rising phase is 0.9236, that is, R² = 0.9236. Meanwhile, the R² of the fast-rising stage fit was 0.9946 and the value of D was 1.29. From an overall perspective, the results of the curve-fitting are reliable (i.e., R² > 0.9).

### Analysis of hydrological response to outflow threshold

**Figure 13:**
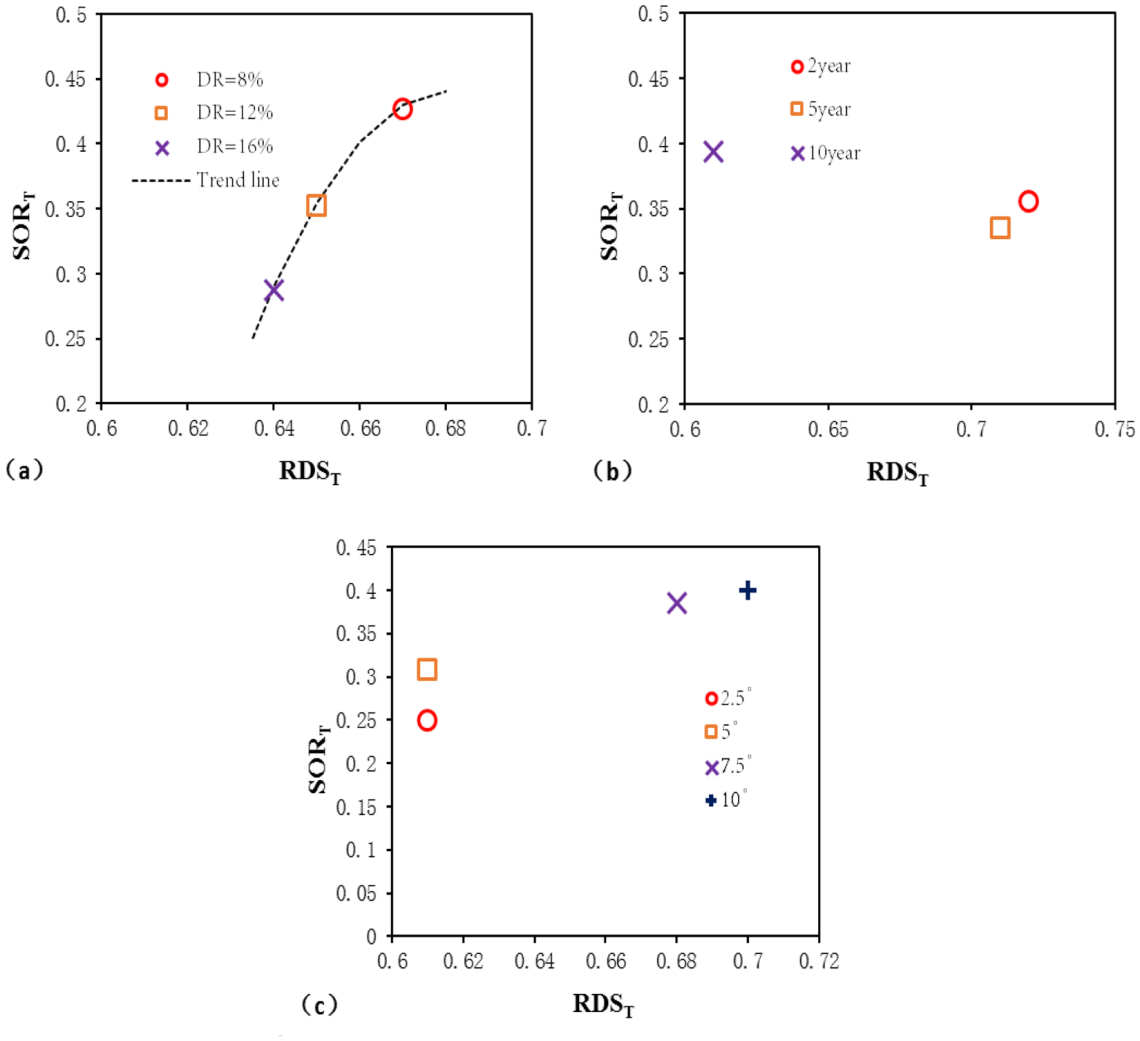
Changes in SOR_T_ and RDS_T_ under the influence of different hydrological factors: (**a**) depression rate; (**b**) rainfall return period; (**c**) slope.

To explore the influence of single hydrological factors on the OT, we continued to adopt the idea of homogenization. One hydrological factor was selected each time as the research object, the results of the remaining hydrological variables were averaged, and the final OT values were calculated. The calculated results are summarized in Table 3, and the plots are shown in Fig. 13.

**Table 3:** Outflow threshold points of the RDOF under the influence of different hydrological factors.

Hydrological factors		Outflow threshold point	
		SOR_T_	RDS_T_
Depression rate (%)	8	0.427	0.67
	12	0.352	0.65
	16	0.287	0.64
Rainfall return period (years)	2	0.356	0.72
	5	0.335	0.71
	10	0.394	0.61
Slope (°)	2.5	0.25	0.61
	5	0.308	0.61
	7.5	0.385	0.68
	10	0.4	0.7

**Figure 14:**
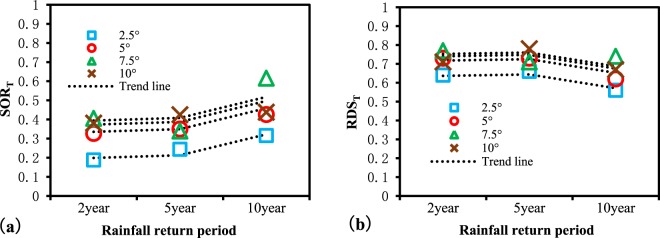
Changes in the outflow threshold point under the influence of different slopes and rainfall return periods: (**a**) SOR_T_ and (**b**) RDS_T_.

It can be seen from Fig. 13 that except for the changes in the OTs under different depression rates, which show a tendency to increase with an increase in DR (Fig. 13a), the results of homogenization under different rainfall return periods and slopes are all relatively heterogeneous (i.e., scattered), although some clustering can be observed. This implies that analyses conducted under complete homogenization may mask some of the underlying patterns of the phenomenon. Therefore, changes observed in the OT under different rainfall return periods and slopes were further analyzed, and the calculation results of the OTs using the average depression rate only are summarized in Table 4 and Fig. 14.

**Table 4:** Outflow threshold points of the RDOF under the influence of different slopes and rainfall return periods.

Slope (°)	Outflow threshold points by rainfall return period					
	2-year		5-year		10-year	
	SOR_T_	RDS_T_	SOR_T_	RDS_T_	SOR_T_	RDS_T_
2.5	0.188	0.64	0.244	0.66	0.316	0.56
5	0.327	0.73	0.35	0.73	0.426	0.62
7.5	0.406	0.77	0.339	0.71	0.616	0.74
10	0.381	0.71	0.425	0.78	0.438	0.67

**Figure 15:**
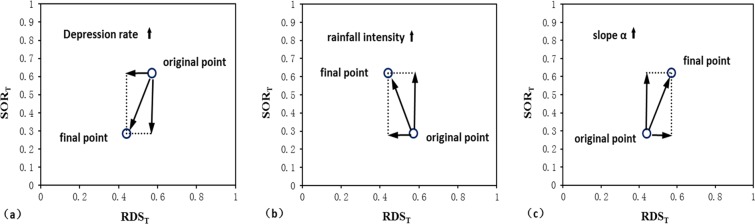
Diagrammatic sketch of changes in the outflow threshold point under the influence of different hydrological factors: (**a**) depression rate; (**b**) rainfall intensity; (**c**) slope.

Although the OTs of varying rainfall intensities and slopes exhibit little regularity, certain overall tendencies can be seen. To fully display these tendencies, trend lines were drawn, as shown in Fig. 14. It can be seen that obvious changes in the OT occurred when the slope increased from 2.5° to 5° and when the rainfall return period increased from 5 to 10 years. These findings demonstrate the nonlinear influence of each hydrological factor on the OT. For a more comprehensible visualization, OT changes under the influence of different hydrological factors were integrated, as shown in Fig. 15.

From Fig. 15, the impacts of the three hydrological factors (i.e., depression rate, rainfall intensity, and slope) on the OT can be seen to vary in direction. The magnitude of these impacts on the changes in SOR_T_ was also greater than for RDS_T_. It is implied that the selection of homogenized RDOF parameters exhibits less deviation for RDS_T_ than for SOR_T_.

With the increase in the depression rate, the surface outflow rate and the relative depression storage corresponding to an OT show a decreasing trend. This is due to the increased depression rate, wherein more water is stored by the depression before surface outflow growth, resulting in the reduction of SOR_T_. Also, it will increase the time needed to spillover, which increases the heterogeneity of the rainfall and the uncertainty of the outflow path. Ultimately, this leads to a slightly earlier spillover time in local areas compared to the spillover time of the total areas and causes RDS_T_ to decrease.

With increased rainfall intensity, the surface outflow rate corresponding to the OT shows an increasing trend, while the relative depression storage shows a decreasing trend. This is because the greater rainfall intensity accelerates the processes of surface wetting and outflow, leading to an increase in the surface outflow rate corresponding to the OT. The reason for the decrease in the relative depression storage corresponding to the OT is likely due to the faster outflow rate and reduced infiltration rate resulting from the greater rainfall intensity.

With increases in slope, the surface outflow rate and relative depression storage corresponding to the OT display an increasing trend. The increased slope accelerates the speed of surface outflow, causing the growth of SOR_T_. However, the increased slope also leads to the reduction of the maximum depression storage, narrowing the difference between the RDS_T_ corresponding to the OT and that of the outflow point of the entire area.

### Analysis of hydrological response to B and D

Homogenization was also used to investigate the hydrological responses of different hydrological factors to parameters B and D in the RDOF. With the application of every hydrological factor, the Levenberg–Marquardt method was used to optimize the values of B and D. The optimization results are summarized in Table 5.

**Table 5:** Parameters of the RDOF under the influence of different hydrological factors.

Hydrological factors		Parameters of RDOF	
		B	D
Depression rate (%)	8	0.7358	0.9877
	12	0.3684	1.2434
	16	0.207	1.5328
Rainfall return period (years)	2	0.5307	0.951
	5	0.4812	1.0727
	10	0.3357	1.3943
Slope (°)	2.5	0.6595	1.089
	5	0.37	1.3107
	7.5	0.4558	1.4262
	10	0.4504	1.2012

In terms of depression rate and rainfall return period, the values of B both show a decreasing tendency with the increase in the depression rate or rainfall intensity. However, changes in the depression rate have a greater impact on B than those of rainfall intensity. The increase in the depression rate from 8% to 12% resulted in a significant change in the value of B (−0.3674), while the increase in the rainfall return period from 2 years (0.824 mm/min) to 10 years (1.209 mm/min) caused a difference in B of only −0.195. For parameter D, the increase in the depression rate and rainfall intensity increased in D. Under the conditions of low depression rate and rainfall intensity, the value of D is less than one, whereas it gradually exceeds one with an increase in the depression rate and rainfall intensity. This is represented graphically by the curve changing from convex to concave, indicating that once spillover occurs, the influence of overflow water on surface outflow will increase.

**Figure 16:**
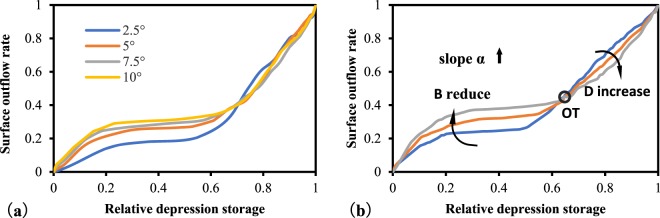
Influence of slope on the shape of the RDOC: (**a**) original; (**b**) diagrammatic sketch.

From the angle of the slope, one point each on B and D is inconsistent with the overall trend. Figures 8 and 16a (which were further homogenized) show that the outliers of B at a slope of 5° may be caused by discretional outflow thresholds of various slopes, which should originally be reduced with increases in slope. The rapid reduction of D at a slope of 10° is likely caused by the existence of more than one outflow threshold in several of the curves. Indeed, the depressions can lead to multiple outflow threshold points under varying terrain conditions. However, for the simplified model, only one outflow threshold was used, and the original value of D should present a trend of gradual enlargement with increases in slope^24^. The overall variation in parameters B and D under different slope conditions are shown in Fig. 16b.

## Conclusions

In this study, the concept of an RDOC was proposed to describe overland flow characteristics under the influence of topographic depressions. The experiments were conducted using a variable-controlled approach with different rainfall return periods, slopes, and depression rates, while ensuring a consistent initial soil moisture content. The function of the RDOC was investigated and the hydrological responses of the function parameters were analyzed.

From our research, we established that the proposed RDOC can reflect overland flow process, the ordinate of which is the relative surface outflow rate, expressed by R_out_/(P-E-i), and it is equivalent to R_out_ / R_yield_ when ignoring surface confinement. An average RDOC was obtained through homogenization. To construct the RDOF, the curve was partitioned into two stages and parameterized by the outflow threshold, which is the reciprocal of the curve index of the first stage (B) and the second stage (D). The function can be integrated into hydrological models as a depression calculation module between the runoff yield and the concentration modules. The complete calculation procedure of the RDOF applied in a hydrological model is provided as part of this work, and the hydrological responses to the function parameters of different hydrological factors and parameter estimation were also performed.

This study provides a new method for analyzing overland flow process under the influence of topographic depressions. The proposed RDOF conveniently accounts for depressions when simulating hydrological processes. However, although we are confident with our results, a limited number of experiments were performed, and therefore more studies are required to confirm our findings.
